# The Metabolic Vulnerability Index (MVX) in Subclinical Thyroid Disorders and Euthyroidism: A Cross-Sectional Exploratory Analysis from the ELSA-Brasil Study

**DOI:** 10.3390/metabo15090606

**Published:** 2025-09-11

**Authors:** Carolina Castro Porto Silva Janovsky, Vandrize Meneghini, William Tebar, Joao Roberto Maciel Martins, José Augusto Sgarbi, Patrícia de Fatima dos Santos Teixeira, Itamar de Souza Santos, Steven R. Jones, Michael J. Blaha, Peter P. Toth, Marcio S. Bittencourt, Raul D. Santos, Paulo A. Lotufo, Layal Chaker, Isabela M. Bensenor

**Affiliations:** 1 Center for Clinical and Epidemiological Research, Universidade de Sao Paulo (USP), São Paulo 05508-000, SP, Brazil; vandrize@gmail.com (V.M.); tebar@usp.br (W.T.); itamarss@usp.br (I.d.S.S.); palotufo@usp.br (P.A.L.); isabensenor@gmail.com (I.M.B.); 2 Division of Endocrinology, Escola Paulista de Medicina/Universidade Federal de São Paulo (UNIFESP), São Paulo 04022-001, SP, Brazil; joao.martins@unifesp.br; 3 Division of Endocrinology, Faculdade de Medicina de Marília, Marília 17519-030, SP, Brazil; jose.sgarbi@gmail.com; 4 Medicine School, Federal University of Rio de Janeiro, Rio de Janeiro 21044-020, RJ, Brazil; pfatima@hucff.ufrj.br; 5 Johns Hopkins, Ciccarone Center for the Prevention of Heart Disease, Baltimore, MD 21287, USA; sjones64@jhmi.edu (S.R.J.); mblaha1@jhmi.edu (M.J.B.); peter.toth@cghmc.com (P.P.T.); 6 CGH Medical Center, Department of Preventive Cardiology, Sterling, IL 61081, USA; 7 Department of Medicine and Department of Radiology, University of Pittsburgh, Pittsburgh, PA 15261, USA; bittencourtms@upmc.edu; 8 Heart Institute (InCor), University of São Paulo Medical School Hospital, São Paulo 05403-900, SP, Brazil; rauldsf@gmail.com; 9 Hospital Israelita Albert Einstein, São Paulo 05652-900, SP, Brazil; 10 Department of Internal Medicine and Rotterdam Thyroid Center, Erasmus Medical Center, 3000 CA Rotterdam, The Netherlands; l.chaker@erasmusmc.nl; 11 Department of Epidemiology, Erasmus University Medical Center, 3000 CA Rotterdam, The Netherlands

**Keywords:** subclinical hypothyroidism, metabolic vulnerability index, inflammation, malnutrition, nuclear magnetic resonance, ELSA-Brasil

## Abstract

Background: Recently, a new biomarker index that reflects inflammation and protein energy malnutrition has emerged as a predictor of mortality in cardiovascular diseases. The metabolic vulnerability index (MVX) derives from blood-based inflammation (IVX) and malnutrition (MMX) markers measured by nuclear magnetic resonance (NMR) spectroscopy. We aimed to explore the association of subclinical hypothyroidism and thyroid-related parameters with IVX, MMX, and MVX scores. Methods: This cross-sectional study used the baseline data from the Brazilian Longitudinal Study of Adult Health (ELSA-Brasil). Individuals with normal thyroid function and subclinical hypothyroidism were included. Thyroid-related parameters—thyroid-stimulating hormone (TSH), free thyroxine (FT4), free triiodothyronine (FT3), the FT3–FT4 ratio, and antithyroperoxidase antibodies (TPOAb)—were the explanatory variables. The primary outcomes, MVX, MMX, and IVX scores, were analyzed as continuous variables. Linear regression analyses were performed for both univariate and multivariable models, with sensitivity and subgroup analyses applied to assess robustness. Findings: There were 3979 participants (51.4% female) with a mean age of 51.26 (SD: 9.02) years. After full adjustment for potential confounder variables, FT3 levels [B: −1.37 (−2.43;−0.31) *p* = 0.011] and the FT3–FT4 ratio [B: −0.90 (−1.79;−0.01) *p* = 0.047] were inversely associated with MVX scores. FT3 levels were also inversely associated with IVX [B: −1.32 (−2.39;−0.24) *p* = 0.017]. These results were consistent in euthyroid individuals and those with cardiometabolic diseases. In the sex-stratified analysis, FT3 levels were inversely associated with MVX, MMX, and IVX scores for men. Conclusion: Lower FT3 levels and the FT3–FT4 ratio were associated with a higher metabolic vulnerability in our cohort. Our study sheds light on the importance of metabolic surveillance in these patients, especially for men with cardiometabolic diseases.

## 1. Introduction

Thyroid hormones have long been recognized as key regulators of metabolism, influencing cardiovascular function, lipid and glucose homeostasis, and thermogenesis [1,2,3,4]. More recently, attention has shifted to their role in regulating inflammation and protein turnover, both of which contribute to cardiovascular morbidity and mortality [5,6,7,8]. Mechanistically, thyroid hormones can affect inflammatory processes by modulating cytokine production, immune cell activity, and oxidative stress [9,10]. In states of inadequate thyroid hormone levels—such as subclinical or overt hypothyroidism—reduced metabolic clearance of inflammatory mediators, altered expression of inflammatory genes, and increased oxidative stress may combine to foster a more proinflammatory environment [11].

Beyond inflammation, thyroid hormone status has a substantial influence on protein metabolism, which is crucial in preventing malnutrition and preserving lean body mass [12,13,14]. Triiodothyronine (T3), the most biologically active thyroid hormone, plays a vital anabolic role by promoting protein synthesis and muscle maintenance [15,16,17,18]. Persistently low T3 levels—sometimes referred to as “low T3 syndrome”—can impair these anabolic processes, leading to reduced muscle mass and weakness. Because strength depends not only on muscle bulk but also on intact neuromuscular transmission and contractile mechanisms, thyroid hormone deficiency may compromise both muscle protein synthesis and the neuromuscular apparatus, contributing to fatigue and decreased physical performance [15,19]. Such deficits exacerbate cardiovascular risk by fostering frailty, reducing metabolic resilience, and weakening immune responses [5,20]. Notably, these mechanisms may operate even in subclinical thyroid disorders, wherein overt hormonal abnormalities are subtle but can still disrupt normal physiologic processes [11].

Taken together, these thyroid hormone-mediated alterations in inflammation and protein status provide mechanistic links to an increased risk of adverse cardiovascular outcomes [10,21]. Chronic, low-grade inflammation and catabolic states—both of which may be intensified by thyroid dysfunction—are increasingly recognized as major contributors to atherosclerotic progression, myocardial remodelling, and heart failure [22,23].

Recently, a new index that associates inflammation and protein malnutrition has emerged as a mortality biomarker in cardiovascular diseases [24,25]. It is the metabolic vulnerability index (MVX), and it derives from the combination of two other indexes: the inflammatory vulnerability index (IVX), which includes markers of inflammation (Glyc-A and small-HDL particles) [21,26], and the metabolic malnutrition index (MMX), which includes malnutrition markers, such as branched-chain amino acids (BCAAs) and citrate levels [27]. All these metabolites are measured by nuclear magnetic resonance (NMR) spectroscopy [28,29]. Therefore, these novel tools that simultaneously capture inflammation and malnutrition biomarkers offer a promising avenue to elucidate how variations in thyroid function and hormone levels translate into clinically meaningful cardiovascular risk. By integrating indicators of both inflammatory and protein–energy malnutrition pathways, MVX may help identify patients whose subtle thyroid-related metabolic imbalances predispose them to greater cardiovascular complications and mortality [15,20].

An important precursor to this work is defining subclinical hypothyroidism and how it is diagnosed. Subclinical hypothyroidism is characterized by a mildly elevated thyroid-stimulating hormone (TSH) concentration in the presence of free thyroxine (FT4) levels within the reference range. Although overt symptoms are absent, this state has been linked to dyslipidaemia, hypertension, and weight gain, and recent guidelines discuss whether or not to treat it [1,11]. Conversely, euthyroidism denotes normal TSH and FT4 levels. In addition to TSH and FT4, the free triiodothyronine (FT3) to FT4 ratio reflects peripheral deiodinase activity and the conversion of the prohormone T4 into the active hormone T3. A low FT3–FT4 ratio may signal reduced tissue conversion and could be a sensitive marker of early thyroid dysfunction even when FT3 and FT4 concentrations remain within their respective reference ranges [30,31]. Our study therefore explored whether these subclinical alterations in thyroid function are mirrored by changes in the MVX and its components.

To date, this index has not been studied in the context of thyroid disorders. Therefore, in this exploratory study, we aimed to investigate the association of subclinical hypothyroidism and thyroid-related parameters with the metabolic vulnerability indexes.

## 2. Methods

### 2.1. Design and Participants

Reporting of this cross-sectional study followed the STROBE (Strengthening the Reporting of Observational Studies in Epidemiology) guidelines for cross-sectional studies [32].

**Figure 1:**
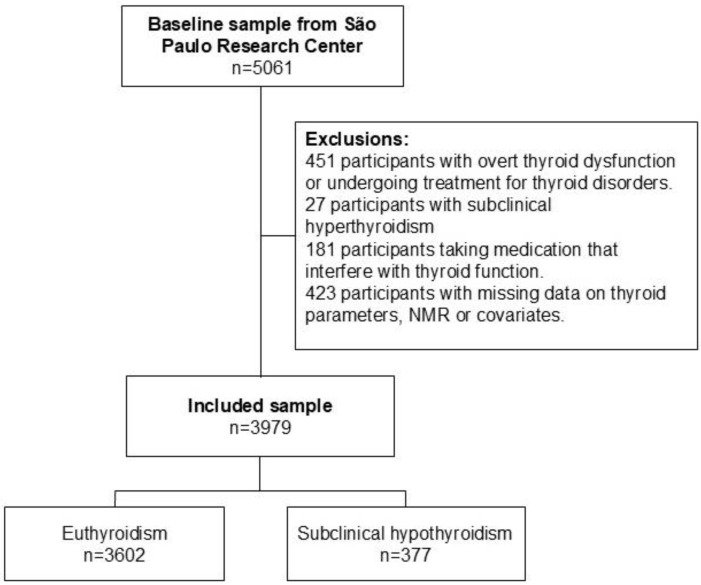
Flowchart of the study sample.

This is a cross-sectional design study using the baseline data of the Brazilian Longitudinal Study of Adult Health (ELSA-Brasil). The ELSA-Brasil represents a large-scale, multicenter, and prospective cohort investigation that has tracked a cohort of middle-aged and older adults residing in 6 state capitals in Brazil [33,34]. The study protocol was standardized and approved in the Ethics Board Committees of all research centres, and individual written consent was obtained from all study participants (Plataforma Brasil CAAE number 08109612.7.1001.0076). The study was conducted in accordance with the Declaration of Helsinki as revised in 2013. The baseline data collection phase (2008–2010) encompassed a comprehensive health evaluation, involving an array of laboratory assays and medical imaging procedures [33,34,35,36,37]. Active and retired civil servants (ranging from 35 to 74 years) were eligible to take part in the study. In this particular analysis, we focused on the subset of participants from the São Paulo research centre of ELSA-Brasil (n = 5061), for whom Nuclear Magnetic Resonance (NMR) data was available at the study’s baseline. Contrary to the notion that the cohort originates from a single deprived community, ELSA-Brasil recruits public servants from universities, research institutes, and healthcare facilities. Consequently, the São Paulo research centre includes individuals from diverse neighbourhoods and socioeconomic backgrounds within the metropolitan area. After applying some exclusion criteria (Figure 1), such as 451 participants with clinical thyroid diseases or taking levothyroxine, propylthiouracil, and thiamazole for the sample, 27 participants with subclinical hyperthyroidism, 181 participants using medications that can alter thyroid function (amiodarone, carbamazepine, carbidopa, furosemide, haloperidol, heparin, levodopa, lithium, metoclopramide, phenytoin, propranolol, primidone, rifampicin, valproic acid, and systemic steroids), and 423 participants with missing data on thyroid or NMR parameters or covariates, the final sample comprised 3979 individuals. There were no systematic differences between participants that were excluded due to missing data (n = 423) from the included sample (n = 3979) (Supplementary Table S1).

### 2.2. MVX, MMX and IVX Measurement

Blood was collected from participants after 8–12 h of nocturnal fasting. The samples were centrifuged at the sites and stored in tubes at −80 °C. GlycA, small high-density lipoprotein particles (S-HDLP from 7.4 nm to 8.7 nm), the branched-chain amino acids (valine, leucine, and isoleucine), and citrate were measured by NMR spectroscopy (LipoProfile^®^ 4 test spectra, LabCorp, Raleigh, NC, USA). GlycA is an aggregate measure of multiple acute-phase inflammatory proteins using the NMR signal of their N-acetyl methyl group protons on N-acetylglucosamine (GlcNAc) moieties located on their bi-, tri-, or tetra-antennary branches (LabCorp, Raleigh, NC, USA) [38,39]. Using NMR, HDL was classified into 7 subspecies that were aggregated into small HDL particles (H1P and H2P), medium HDL particles (H3P and H4P), and large HDL particles (H5P to H7P) [40]. GlycA and small HDL particles were combined in the inflammatory vulnerability index (IVX). BCCA and citrate were combined in the metabolic malnutrition index (MMX). Sex-specific MVX, MMX, and IVX scores were calculated using the software algorithm previously reported (Supplementary Table S2). To enhance interpretability, the final scores were standardized using z-score transformation and should be interpreted per 1 standard deviation. MVX was designated as the primary outcome, while MMX and IVX served as secondary outcomes.

### 2.3. Thyroid-Related Parameters

Venous blood samples were drawn in the morning after an overnight fast (6:30 AM to 9:00 AM). TSH, FT4, and FT3 were determined by a third-generation immunoenzymatic assay (Roche Diagnostics, Manheim, Germany). The analysis included euthyroid participants (TSH levels from 0.40 to 4.00 mIU/L with no history of levothyroxine or anti-thyroid drug use) and subclinical hypothyroidism (TSH levels > 4.00 mIU/L with FT4 levels from 0.93 to 1.70 ng/dL and no use of thyroid drugs). The FT3–FT4 ratio is an indicator of peripheral thyroid hormone metabolism, reflecting how effectively the prohormone (T4) is being converted into the biologically active hormone (T3) in the body’s tissues. Thus, it allows us to explore subtle or subclinical changes in thyroid function that may not be fully captured by looking at free T3, free T4, or TSH levels alone [36,37,38]. Serum antithyroperoxidase antibody (TPOAb) levels were determined by electrochemiluminescence (Roche Diagnostics, Mannheim, Germany), and values ≥34.00 IU/mL were considered positive.

### 2.4. Other Baseline Variables

Sociodemographic data, risk factors, and health conditions were investigated through questionnaires and laboratory measures [34]. The questionnaires addressed age (in years), sex (male/female), self-reported race (non-white/white), smoking status (never/past/current), alcohol intake (no/yes), family history of CVD (no/yes), and presence of coronary heart disease (no/yes). Physical activity during leisure time was assessed using the International Physical Activity Questionnaire (IPAQ) and classified as inactive (<10 min/week of moderate/vigorous physical activity or walking); not sufficiently active (<75 min/week in vigorous or <150 min/week of walking and moderate physical activity or any combination); and active (≥75 min/week in vigorous or ≥150 min/week of moderate physical activity or walking and/or any combination). Diet was quantified based on adherence to a Mediterranean Eating Pattern for Americans (MEPA) diet pattern, according to the cardiovascular health metric used in the Life’s Essential 8 [41]. Higher scores (≥50 points) represented higher adherence to an ideal diet, while lower scores (<50 points) indicated lower adherence. Participants reported the use of medication in the two weeks prior to the interview. Body mass index (BMI in kg/m^2^) was calculated through the following equation: BMI = weight in kg/height in m^2^. We defined hypertension (no/yes) as the use of antihypertensive drugs, systolic blood pressure ≥140 mm Hg, or diastolic blood pressure ≥90 mm Hg. Diabetes was defined (no/yes) as a previous medical diagnosis of diabetes, use of anti-diabetic medications (no/yes), a fasting plasma glucose ≥126 mg/dL (7.0 mmol/L), a 2 h plasma glucose after a glucose overload ≥200 mg/dL (11.1 mmol/L), or a glycated hemoglobin ≥6.5% (48 mmol/mol). Dyslipidemia was defined as a previous medical history of dyslipidemia, use of lipid lowering medication, or presented LDL-cholesterol ≥130 mg/dL after a 12 h fast. The estimated glomerular filtration rate (eGFR in mL/min per 1.73 m^2^) was calculated with the 2021 CKD-EPI equation. The presence of cardiometabolic diseases (yes) comprised having at least one of the following diseases: diabetes, dyslipidemia, hypertension, obesity (BMI ≥ 30 kg/m^2^), and coronary heart disease.

These behavioural and clinical characteristics were selected because they are established cardiovascular risk factors and can influence both thyroid function and systemic inflammation. Including them as covariates in our analyses helps to reduce confounding and better isolate the associations between thyroid parameters and MVX, IVX, and MMX scores.

### 2.5. Data Analyses

Data were presented as counts (n) and percentages (%) or means and standard deviations (SD). The comparison of the sociodemographic variables (age, race, and sex), risk factors, and health conditions (BMI, eGFR, diabetes, hypertension, dyslipidemia, coronary heart disease, family history of CVD, alcohol intake, physical activity, diet, and smoking) according to the thyroid function was performed using chi-square/Fisher’s exact tests and independent sample *t*-test for categorical and continuous variables, respectively. The distribution of continuous variables was assessed with the Shapiro–Wilk test, and variables showing significant skewness were normalized using a natural logarithmic transformation. For parameters that could theoretically assume the value zero (such as TSH in some participants), a small constant was added before transformation [ln(x + 1)] to avoid taking the logarithm of zero. This approach improved normality and allowed parametric analyses. Associations between thyroid-related parameters (exposures) and MVX, IVX, and MMX z-scores (outcomes) were assessed using linear regression models. Results are presented as beta coefficients (B) with 95% confidence intervals (95% CI) for both univariate and multivariate models. Multivariate adjustments were applied as follows:

- Model 1: Adjusted for age, sex, race, smoking, diabetes, hypertension, BMI, dyslipidemia, estimated glomerular filtration rate, previous coronary heart disease, and family history of cardiovascular disease.
- Model 2: Further adjusted for physical activity, alcohol intake, and diet.

To account for potential non-linear associations, a quadratic term for each thyroid parameter was included in Model 2. Sensitivity analyses were conducted for euthyroid individuals, and subgroup analyses were performed by sex and the presence of cardiometabolic diseases. In sex-stratified analyses, standardized values of thyroid-related parameters were used to facilitate comparison. Model fit was evaluated using Bayesian Information Criteria (BIC) and log-likelihood, with improvements observed in Model 2. To address multiple comparisons, we applied Bonferroni, Holm, and Benjamini–Hochberg (BH) corrections to the *p*-values from 18 multivariate models (3 outcomes × 6 exposures); corrected *p*-values are provided in Supplementary Table S3. All statistical analyses were performed using IBM SPSS Statistics v.30, with a significance level set at *p* < 0.05.

## 3. Results

The mean age of participants (n = 3979) was 51.26 ± 9.02 years; 51.4% were female, and 58.6% self-identified as white. Subclinical hypothyroidism was prevalent in 9.5% (n = 377) of the individuals. Participants with subclinical hypothyroidism were older, had a lower proportion of participants classified as non-white (which in our study grouped self-reported black, brown, Asian, and indigenous categories), a higher BMI, and a lower glomerular filtration rate than the euthyroid participants (*p* < 0.05). Individuals with subclinical hypothyroidism presented a higher prevalence of positive TPOAb (22.0% vs. 8.0%) and greater MMX scores (51.21 ± 6.03 vs. 50.43 ± 6.02, *p* = 0.017) when compared to euthyroid. Detailed characteristics of participants are presented in Table 1.

Table 2 shows thyroid-related parameters associated with the MVX, IVX, and MMX scores. Free T4, free T3, the FT3–FT4 ratio, and positive TPOAb showed statistically significant associations with MVX scores in the crude analyses. After adjustment for sociodemographic variables, cardiovascular risk factors, and health conditions, FT3 levels [B: −1.37 (−2.43; −0.31) *p* = 0.011] and FT3–FT4 ratio [B: −0.90 (−1.79;−0.01) *p* = 0.047] were inversely associated with MVX scores. Associations of the FT3–FT4 ratio and positive TPOAb lost significance after adjustment (*p* > 0.05). Subclinical hypothyroidism [B: 0.13 (0.02; 0.23) *p* = 0.017], FT3 [B: −4.05 (−5.13;−2.97) *p* < 0.001], FT3–FT4 ratio [B: −2.27 (−3.21;−1.32) *p* < 0.001], and positive TPOAb [B: 0.12 (0.01;0.23) *p* = 0.029] were associated with the MMX scores in the crude analysis. However, no association remained statistically significant after the multivariable adjustment. Regarding the inflammatory score (IVX), we verified an inverse association of FT4 [B: −0.31 (−0.49;−0.12) *p* = 0.001] and FT3 [B: −2.91 (−3.99;−1.83) *p* < 0.001] levels with IVX scores. After the adjustments, only the log-transformed levels of FT3 remained inversely associated with IVX [B: −1.32 (−2.39;−0.24) *p* = 0.017] (Table 2). When we applied multiple comparison corrections to the *p*-values in Model 2, none of the associations remained statistically significant (*p* > 0.05) (Supplementary Table S3).

Furthermore, we restricted the analyses to include only euthyroid individuals (n = 3602). The results showed that FT3 levels and the FT3–FT4 ratio were statistically significantly associated with MVX (FT3, B: −1.62 (−2.75; −0.50) *p* = 0.004; FT3–FT4 ratio, B: −1.10 (−2.04; −0.16) *p* = 0.022) and IVX (FT3, B: −1.61 (−2.75; −0.47) *p* = 0.006; FT3–FT4 ratio, B: −1.05 (−2.01; −0.09) *p* = 0.032) (Table 2).

**Figure 2:**
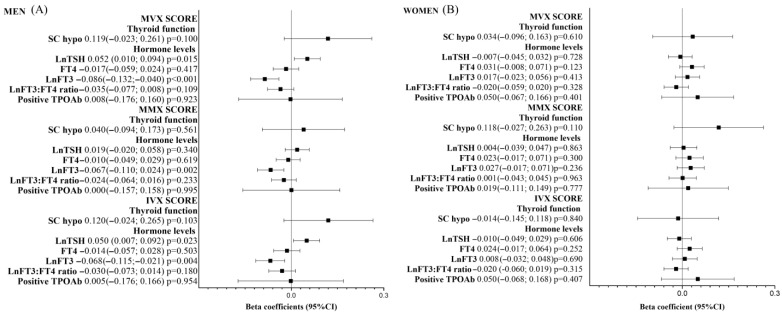
Association of standardized thyroid-related parameters with MVX, MMX, and IVX z-scores for (**A**) men (n = 1933) and (**B**) women (n = 2046).

Subgroup analyses using sex-stratified data showed positive associations of TSH with MVX and IVX scores and an inverse association of lnFT3 with MVX, MMX, and IVX scores only for men (Figure 2A). There was no significant association between thyroid-related parameters and MVX, MMX, and IVX scores for women (Figure 2B).

To better understand the impact of cardiometabolic diseases on the associations of thyroid markers with MVX, MMX, and IVX scores, we re-analyzed the sample according to the absence or the presence of at least one cardiometabolic disease (diabetes, dyslipidemia, hypertension, obesity, and coronary heart disease). The results showed that the associations of FT3 and FT3–FT4 ratio with MVX (FT3, B: −1.56 (−2.86;−0.25) *p* = 0.019; FT3–FT4 ratio, B: −1.12 (−2.19;−0.06) *p* = 0.039) and IVX (FT3, B: −1.91 (−3.24;−0.58) *p* = 0.005; FT3–FT4 ratio, B: −1.31 (−2.39;−0.22) *p* = 0.019) remained statistically significant only for the group with at least one cardiometabolic disease (Supplementary Table S4).

### Non-Linear Associations

In the non-linear analysis, significant quadratic relationships were observed, indicating U-shaped associations between the FT3–FT4 ratio and MMX scores in the main analysis [B: −13.44 (−25.07;−1.81) *p* = 0.024], for euthyroid individuals [B: −12.74 (−24.58;−0.90) *p* = 0.035], and for men [B: −48.11 (−70.66;−25.57) *p* < 0.001]. These findings indicate that both low and high FT3–FT4 ratios were associated with higher MMX scores, with the strongest effect observed in men. Additionally, in men, significant U-shaped associations were found between the FT3–FT4 ratio and MVX [B: −27.99 (−52.13;−3.85) *p* = 0.023], FT3 levels and MVX [B: −84.34 (−131.01;−37.66) *p* < 0.001], and FT3 levels and IVX [B: −87.25 (−134.81;−39.70) *p* < 0.001] (Supplementary Table S5; Supplementary Figure S1). All significant quadratic models demonstrated better fit than models containing only the linear term, further supporting the presence of non-linear associations.

## 4. Discussion

Our study aimed to investigate the relationship between thyroid-related parameters and the metabolic vulnerability index (MVX), a composite measure reflecting inflammation (IVX) and malnutrition (MMX) components. Our primary findings demonstrated a negative association between FT3 levels and the FT3–FT4 ratio with MVX scores. Notably, IVX emerged as the primary contributor to MVX, suggesting that inflammation may play a more significant role than malnutrition in determining metabolic vulnerability in individuals with thyroid disorders and also in euthyroid participants. Although statistical significance was lost after correcting for multiple comparisons, these associations were consistent in subgroup analyses for euthyroid individuals, men, and those with cardiometabolic diseases. Additionally, we observed a non-linear relationship between the FT3–FT4 ratio and MMX scores in the overall analysis, as well as in euthyroid individuals and men.

In previous recent analyses, the metabolic vulnerability index (MVX) was described as a mortality marker that encompasses inflammation and protein–energy wasting parameters [24,25]. Otvos et al. were the first to acknowledge that this index could measure this metabolic malnutrition-inflammation syndrome, which is intimately related to survival in the two large cardiac catheterization cohorts [24]. In this study, MVX exhibited a strong association with mortality, independently of sex, age, or comorbidities [24]. Similarly, Conners et al. found a significant association of MVX and mortality in a heart failure community cohort independently of traditional risk factors, thus underscoring the potential use of MVX to stratify risk in these patients [25]. The subtle metabolic disturbances observed in subclinical hypothyroidism might not manifest through traditional cardiovascular risk factors but may still affect inflammation and protein–energy balance. By simultaneously capturing biomarkers of inflammation and malnutrition, MVX offers a novel opportunity to elucidate how variations in thyroid function and hormone levels translate into clinically meaningful cardiovascular risk. Integrating indicators of both inflammatory and protein–energy malnutrition pathways may help identify patients whose subtle thyroid-related metabolic imbalances predispose them to greater cardiovascular complications and mortality [15,20].

When we further analyzed the metabolic vulnerability index, we could decompose the index into two parameters: the inflammation vulnerability index (IVX), which reflects metabolic parameters associated with cardiovascular risk as small high density lipoprotein particles (SHDLP) and Glyc-A, and the metabolic malnutrition index (MMX), which includes branched-chain amino acids (leucine, isoleucine, and valine) and citrate, reflecting nutrition status and levels of sarcopenia and cachexia [42,43,44,45,46]. GlycA is a measure of the glycan residues of several acute phase glycoproteins and reflects the intensity of systemic inflammation [36,47,48,49], while SHDLP facilitates protective functions of anti-inflammatory and immune response proteins [46,50]. BCAA and citrate have been scarcely studied as malnutrition markers in heart failure [51,52] and even more scarcely in thyroid disorders [27,53,54]. In fact, amino acids in thyroid dysfunctions are mostly associated with thermoregulatory impairment rather than malnutrition, as they are usually not consumptive diseases [53,54]. Our study has shown that the inflammatory index (IVX) was more consistent with the results from MVX, showing an inverse association with free T3 levels, even when the analysis was restricted to euthyroid participants. On the other hand, the metabolic malnutrition index (MMX) was only significantly associated with the FT3–FT4 ratio.

Current clinical guidelines recommend levothyroxine therapy primarily for overt hypothyroidism or subclinical hypothyroidism with a TSH concentration above 10 mIU/L, the presence of anti-thyroid antibodies, or clear symptoms [1,11]. The exploratory associations observed in our study do not warrant changes to these recommendations, but they imply that metabolic vulnerability may already be increased in euthyroid patients or those with mild TSH elevations. Until longitudinal studies clarify whether intervention based on MVX improves outcomes, clinicians should continue to follow guideline-directed therapy while maintaining vigilance for cardiometabolic risk factors in individuals with subtle thyroid hormone variations. When we further analyzed the metabolic vulnerability index, we could decompound the index into two parameters: the inflammation vulnerability index (IVX), which reflects metabolic parameters associated with cardiovascular risk as small high density lipoprotein particles (SHDLP) and Glyc-A, and the metabolic malnutrition index (MMX), which includes branched-chain amino acids (leucine, isoleucine, and valine) and citrate, reflecting nutrition status and levels of sarcopenia and cachexia [42,43,44,45,46]. GlycA is a measure of the glycan residues of several acute phase glycoproteins and reflects the intensity of systemic inflammation [36,47,48,49], while SHDLP facilitates protective functions of anti-inflammatory and immune response proteins [46,50]. BCAA and citrate have been scarcely studied as malnutrition markers in heart failure [51,52] and even more scarcely in thyroid disorders [27,53,54]. In fact, amino acids in thyroid dysfunctions are mostly associated with thermoregulatory impairment rather than malnutrition, as they are usually not consumptive diseases [53,54]. Our study has shown that the inflammatory index (IVX) was more consistent with the results from MVX, showing an inverse association with free T3 levels, even when the analysis was restricted to euthyroid participants. On the other hand, the metabolic malnutrition index (MMX) was only significantly associated with the FT3–FT4 ratio.

Compared to the previous studies, in the present analysis the inverse association of FT3 and the FT3–FT4 ratio was only found in men but not in women. Interestingly, we observed that the associations between thyroid-related parameters and the scores were restricted to the male sex after stratifying the data. The possible explanations for that result could be immune function regulation by sexual hormones’ modulation: testosterone may have different effects on immune responses and inflammation compared to estrogen [55,56,57]. Also, differences in body composition, fat distribution, and muscle mass between sexes can influence metabolic processes and the body’s response to thyroid hormones [58,59,60,61,62,63]. In addition, recent studies that evaluate the association of TPOAb showed an association with all-cause, cardiovascular, and cancer mortality in men but not in women [64,65]. Moreover, the burden of hypothyroidism in men in Brazil appears to be higher than in the classic thyroid studies [66,67,68,69,70]. In fact, in a population-based study in a very deprived area in the state of São Paulo, the ratio of women to men was 1.1 for overt and subclinical hypothyroidism [67]. All these possible explanations might make the associations between thyroid parameters and inflammatory and nutritional status more apparent in males.

Although TPOAb positivity was more frequent in subclinical hypothyroidism, TPOAb was not independently associated with MVX, IVX, or MMX after multivariable adjustment and remained non-significant after Benjamini–Hochberg correction. This null association suggests that these NMR-derived indices chiefly reflect the peripheral thyroid hormone milieu—especially FT3 and the FT3–FT4 ratio—rather than thyroid autoimmunity per se; longitudinal studies with serial TPOAb/TgAb measurements are warranted.

Our study has some limitations. While it provides valuable insights, it is cross-sectional and cannot establish causality. Future longitudinal analyses and randomized controlled trials are needed to further explore these associations and the potential benefits of therapeutic interventions. In fact, our group is already investigating how these indexes perform in predicting cardiovascular and mortality risk in subclinical thyroid disorders in a prospective study. Also, we could not evaluate the individuals with subclinical hyperthyroidism because the number of such participants in our cohort was very low, precluding reliable comparison. As an exploratory analysis, we tested multiple comparisons, which might have led to false positive results. Although the associations between the independent and dependent variables were not statistically significant after applying multiple comparisons corrections, it is noteworthy that these associations remained consistent across both crude and multivariate models, as well as in the sensitivity and subgroup analyses. These findings suggest that the relationships observed may be robust, but further validation in independent cohorts is needed to confirm their true nature. Additionally, the *p*-value correction for multiple comparisons may be overly conservative in our scenario. These techniques rely on the independence among tested hypotheses. In our study, the exposures and outcomes are correlated, usually leading to a lower type I error inflation than assumed by these methods. Therefore, results before *p*-value correction for multiple comparisons should not be overlooked, and the consistency of results across various analytical approaches supports their potential relevance.

Beyond these methodological considerations, several contextual factors should temper interpretation. First, the ELSA-Brasil cohort comprises Brazilian civil servants from urban centres. Although the São Paulo centre includes participants from diverse socioeconomic backgrounds, our findings may not generalize to populations in other geographic regions or to rural settings, where environmental exposures and access to healthcare differ markedly. Also, the MVX, IVX, and MMX scores require measurements of Glyc-A, small HDL particles, and amino acids by nuclear magnetic resonance spectroscopy. This technology is costly and not yet widely available in routine clinical practice, limiting the immediate clinical applicability of our findings and underscoring the need for cost-effectiveness analyses before implementation in routine care.

Our study has several strengths as well. This was the first study to examine the association of thyroid-related parameters and the metabolic vulnerability index. This index was associated with mortality risk in previous studies [24,25], and it needs to be confirmed in other populations, including ELSA-Brasil’s. All other MVX cohorts in previous analyses were from high-income countries, and this study included a sample from Brazil, a low–middle-income country. Our results about the association of thyroid parameters and MVX must be replicated in other cohorts with information about thyroid function.

In conclusion, we found an inverse association of low FT3 levels and the FT3–FT4 ratio with MVX and IVX but not with MMX. The findings suggest a higher risk of inflammation and metabolic vulnerability in the individuals with lower levels of FT3 and the FT3–FT4 ratio, especially for men and those with cardiometabolic diseases. Furthermore, the impact of the FT3–FT4 ratio on MMX appears to be significant only for men.

## 5. Conclusions

We found an inverse association of low FT3 levels and the FT3–FT4 ratio with MVX and IVX scores but not with MMX. Individuals—particularly men and those with cardiometabolic diseases—with lower FT3 and FT3–FT4 ratios exhibited higher metabolic vulnerability, driven mainly by inflammation. Conversely, the influence of the FT3–FT4 ratio on MMX appeared to be significant only for men. These findings underscore the importance of monitoring metabolic and inflammatory profiles in the context of thyroid function. If validated in future cohorts, the MVX could become a useful tool for clinicians and public health practitioners to identify individuals at heightened cardiometabolic risk and tailor preventive strategies. Future studies should evaluate whether incorporating MVX into routine clinical evaluation improves risk prediction and explore interventions—either pharmacologic or lifestyle—that might favourably modify MVX scores. Our analyses are exploratory and cross-sectional; without prospective outcome data we cannot infer causality. Thus, the present results should be viewed as hypothesis-generating and require validation in longitudinal studies before any definitive recommendations can be made.

## Figures and Tables

**Table 1:** Demographics, risk factors, and health conditions of all baseline sample and according to thyroid function.

	All Sample (n = 3979)	Thyroid Function		*p*-Value
		Euthyroid (n = 3602)	Subclinical Hypothyroidism (n = 377)	
**Sociodemographic variables**				
**Age**, years	51.26 (9.02)	51.11 (8.99)	52.72 (9.16)	**0.001**
**Sex**				0.816
Male	1933 (48.6%)	1752 (48.6%)	181 (48.0%)	
Female	2046 (51.4%)	1850 (51.4%)	196 (52.0%)	
**Self-reported race**				**0.021**
Non-white	1646 (41.4%)	1511 (41.9%)	135 (35.8%)	
White	2333 (58.6%)	2091 (58.1%)	242 (64.2%)	
**Risk factors and health conditions**				
**BMI**, kg/m^2^	27.27 (4.87)	27.21 (4.81)	27.82 (5.41)	**0.038**
**eGFR**, mL/min per 1.73 m^2^	85.28 (15.05)	85.64 (15.06)	81.83 (14.51)	**<0.001**
**Smoking**				0.071
Never	2125 (53.4%)	1907 (52.9%)	218 (57.8%)	
Past/current	1854 (46.6%)	1695 (47.1%)	159 (42.2%)	
**Alcohol intake**				0.134
No	2166 (54.4%)	1947 (54.1%)	219 (58.1%)	
Yes	1813 (45.6%)	1655 (45.9%)	158 (41.9%)	
**Physical Activity**				0.412
Inactive	2603 (65.4%)	2347 (65.2%)	256 (67.9%)	
Not sufficiently active	454 (11.4%)	410 (11.4%)	44 (11.7%)	
Active	922 (23.2%)	845 (23.5%)	77 (20.4%)	
**Diet**				0.162
Lower (score < 50)	950 (23.9%)	871 (24.2%)	79 (21.0%)	
Higher (score ≥ 50)	3029 (76.1%)	2731 (75.8%)	298 (79.0%)	
**Diabetes**	688 (17.3%)	630 (17.5%)	58 (15.4%)	0.304
**Hypertension**	1232 (31.0%)	1105 (30.7%)	127 (33.7%)	0.229
**Dyslipidemia**	1718 (43.2%)	1556 (43.2%)	162 (43.0%)	0.932
**Coronary heart disease**	96 (2.4%)	86 (2.4%)	10 (2.7%)	0.724 ^a^
**Family history of CVD**	1016 (25.5%)	913 (25.3%)	103 (27.3%)	0.403
**Cardiometabolic disease**	2695 (67.7%)	2433 (67.5%)	262 (69.5%)	0.441
**Positive TPOAb**	371 (9.3%)	288 (8.0%)	83 (22.0%)	<0.001
**MVX**	43.60 (9.09)	43.51 (9.11)	44.42 (8.87)	0.065
**IVX**	40.70 (10.93)	40.64 (10.99)	41.25 (10.42)	0.303
**MMX**	50.51 (6.02)	50.43 (6.02)	51.21 (6.03)	**0.017**

Data are mean (SD) or n (%). CVD: cardiovascular disease. eGFR: estimated glomerular filtration rate. BMI: body mass index. MVX (metabolic vulnerability index), IVX (inflammation vulnerability index). MMX (metabolic malnutrition index). *p*-value: independent sample *t*-test or chi-square. ^a^ Fisher’s exact test.

**Table 2:** Association of thyroid-related parameters with MVX, MMX, and IVX scores in individuals with euthyroid and subclinical dysfunction.

	Crude		Model 1		Model 2		Model 2 Including Only Euthyroid Individuals	
	B (95% CI)	*p*-Value	B (95% CI)	*p*-Value	B (95% CI)	*p*-Value	B (95% CI)	*p*-Value
**MVX SCORE**								
Subclinical hypo	0.10 (−0.01;0.20)	0.065	0.09 (−0.01;0.18)	0.082	0.07 (−0.02;0.17)	0.146	-	-
LnTSH	0.04 (−0.04;0.13)	0.350	0.07 (−0.01;0.15)	0.094	0.06 (−0.02;0.14)	0.137	0.01 (−0.10;0.12)	0.894
FT4	−0.29 (−0.47;−0.10)	0.002	0.02 (−0.16;0.19)	0.853	0.02 (−0.16;0.19)	0.856	0.03 (−0.16;0.21)	0.779
LnFT3	−4.24 (−5.31;−3.17)	<0.001	−1.17 (−2.24;−0.10)	0.033	−1.37 (−2.43;−0.31)	0.011	−1.62 (−2.75;−0.50)	0.004
LnFT3–FT4 ratio	−1.43 (−2.37;−0.49)	0.003	−0.78 (−1.68;0.12)	0.088	−0.90 (−1.79;−0.01)	0.047	−1.10 (−2.04;−0.16)	0.022
Positive TPOAb	0.11 (0.01;0.22)	0.034	0.03 (−0.07;0.13)	0.529	0.03 (−0.07;0.12)	0.596	0.01 (−0.10;0.12)	0.844
**MMX SCORE**								
Subclinical hypo	0.13 (0.02;0.23)	0.017	0.08 (−0.02;0.18)	0.125	0.07 (−0.03;0.17)	0.155	-	-
LnTSH	0.07 (−0.02;0.15)	0.130	0.03 (−0.05;0.11)	0.458	0.03 (−0.05;0.11)	0.517	−0.05 (−0.16;0.06)	0.370
FT4	−0.05 (−0.24;0.13)	0.583	0.05 (−0.13;0.23)	0.574	0.04 (−0.14;0.22)	0.684	0.06 (−0.13;0.25)	0.534
LnFT3	−4.05 (−5.13;−2.97)	<0.001	−0.47 (−1.56;0.62)	0.401	−0.51 (−1.60;0.57)	0.354	−0.51 (−1.66;0.63)	0.380
LnFT3–FT4 ratio	−2.27 (−3.21;−1.32)	<0.001	−0.34 (−1.26;0.57)	0.459	−0.31 (−1.22;0.60)	0.507	−0.40 (−1.36;0.56)	0.412
Positive TPOAb	0.12 (0.01;0.23)	0.029	0.01 (−0.09;0.11)	0.786	0.02 (−0.08;0.12)	0.765	−0.01 (−0.12;0.10)	0.851
**IVX SCORE**								
Subclinical hypo	0.06 (−0.05;0.16)	0.302	0.07 (−0.03;0.17)	0.192	0.05 (−0.05;0.15)	0.304	-	-
LnTSH	0.01 (−0.07;0.10)	0.775	0.06 (−0.02;0.14)	0.136	0.05 (−0.03;0.14)	0.187	0.02 (−0.09;0.13)	0.662
FT4	−0.31 (−0.49;−0.12)	0.001	−0.01 (−0.19;0.17)	0.942	0.00 (−0.18;0.18)	0.999	0.00 (−0.19;0.19)	0.991
LnFT3	−2.91 (−3.99;−1.83)	<0.001	−1.10 (−2.19;−0.01)	0.048	−1.32 (−2.39;−0.24)	0.017	−1.61 (−2.75;−0.47)	0.006
LnFT3–FT4 ratio	−0.53 (−1.47;0.41)	0.268	−0.71 (−1.63;0.20)	0.127	−0.87 (−1.77;0.04)	0.060	−1.05 (−2.01;−0.09)	0.032
Positive TPOAb	0.08 (−0.03;0.18)	0.157	0.03 (−0.07;0.13)	0.527	0.03 (−0.07;0.12)	0.613	0.02 (−0.09;0.13)	0.749

Model 1 adjustment by age, sex, race, smoking, diabetes, hypertension, BMI, dyslipidemia, estimated glomerular filtration rate, and family history of cardiovascular disease. Model 2 added alcohol intake, physical activity, and diet.

## Data Availability

Data will be available upon request.

## Supplementary Materials

The following supporting information can be downloaded at: https://www.mdpi.com/article/10.3390/metabo15090606/s1.
