# Supplementary material for: The Metabolic Vulnerability Index (MVX) in Subclinical Thyroid Disorders and Euthyroidism: A Cross-Sectional Exploratory Analysis from the ELSA-Brasil Study
Source: Metabolites. 2025 Sep 11;15(9):606. doi: 10.3390/metabo15090606 (PMC12471407; doi:10.3390/metabo15090606)
Supplement: Supplementary file 1 [file metabolites-15-00606-s001.zip › metabolites-3741463-supplementary.pdf]

Supplementary Table S1. Demographics, risk factors and health conditions of the sample that had any missing data comparing to the included sample.

	Excluded (n<423)	Included sample (n=3979)	p-value
**Sociodemographic variables**			
**Age**, years	50.52 (9.01)	51.26 (9.02)	0.105
**Sex**			0.889
Male	207 (48.9%)	1933 (48.6%)	
Female	216 (51.1%)	2046 (51.4%)	
**Self-reported race**			0.309
Non-white	161 (44.1%)	1646 (41.4%)	
White	204 (55.9%)	2333 (58.6%)	
**Risk factors and health conditions**			
**BMI**, kg/m^2	27.50 (4.71)	27.27 (4.87)	0.357
**eGFR**, mL/min per 1.73 m^2	86.58 (14.68)	85.28 (15.05)	0.090
**Smoking**			0.270
Never	214 (50.6%)	2125 (53.4%)	
Past / current	209 (49.4%)	1854 (46.6%)	
**Alcohol intake**			0.825
No	231 (55.0%)	2166 (54.4%)	
Yes	189 (45.0%)	1813 (45.6%)	
**Physical Activity**			0.008
Inactive	209 (74.4%)	2603 (65.4%)	
Not sufficiently active	26 (9.3%)	454 (11.4%)	
Active	46 (16.4%)	922 (23.2%)	
**Diet**			0.111
Lower (score <50)	115 (27.4%)	950 (23.9%)	
Higher (score ≥50)	305 (72.6%)	3029 (76.1%)	
**Diabetes**	76 (18.0%)	688 (17.3%)	0.727
**Hypertension**	150 (35.7%)	1232 (31.0%)	0.046
**Dyslipidemia**	190 (45.8%)	1718 (43.2%)	0.308
**Family history of CVD**	78 (24.7%)	1016 (25.5%)	0.738
**MVX**	42.86 (9.30)	43.60 (9.09)	0.129
**IVX**	40.10 (10.88)	40.70 (10.93)	0.302
**MMX**	50.08 (5.64)	50.51 (6.02)	0.179
**TSH levels**	2.39 (1.39)	2.32 (1.40)	0.316
**FT4 levels**	1.20 (0.17)	1.20 (0.17)	0.788

Data are mean (SD) or n (%). CVD: cardiovascular disease. eGFR: estimated glomerular filtration rate. BMI: body mass index. p-value: independent' sample t-test or chi-square.

Supplementary Table S2. Calculations of sex-specific IVX, MMX, and MVX scores.

**Female score equations**	
**IVX**	IVX_F = 9 + (GlycA *-0·000187) + (S-HDLP * -0·3585) + ((GlycA * S-HDLP)* 0·000348)<br>Range: Min=3·0 (score=1); Max=9·0 (score=100)
**MMX**	MMX_F = ((4 + (Leu * -0·03142) + (Leu^2 * 0·0000893)) * 0·353) +<br>((7 + (Val * -0·03362) + (Val^2 * 0·0000689)) * 0·684) + (Ileu * 0·00332) +<br>((1 + (Citr * -0·0072) + ($Citr^2$ * 0·0000573)) * 0·7135)<br>Range: ln Min=0·8 (score=1); ln Max=1·8 (score=100)
**MVX**	MVX_F = (IVX_F * 2·27278) + (lnMMX_F * 12·13511) + (IVX_F * lnMMX_F) * -1·09312<br>Range: Min=17·0 (score=1); Max=25·0 (score=100)
**Male score equations**	
**IVX**	IVX_M = 9 + (GlycA * -0·00437) + (S-HDLP * -0·52307) + ((GlycA * S-HDLP) * 0·000817)<br>Range: Min=1·4 (score=1); Max=7·6 (score=100)
**MMX**	MMX_M = ((4 + (Leu * -0·01594) + (Leu^2 * 0·0000291)) * 1·076) +<br>((7 + (Val * -0·0239) + (Val^2 * 0·00005)) * 0·414) + (Ileu * 0·01265) +<br>((1 + (Citr * 0·00906) + ($Citr^2$ * -0·0000126)) * 0·5881)<br>Range: ln Min=1·59 (score=1); ln Max=2·1 (score=100)
**MVX**	MVX_M = (IVX_M * 3·54601) + (lnMMX_M * 14·41428) + (IVX_M * lnMMX_M) * -1·43438<br>Range: Min=27·0 (score=1); Max=34·2 (score=100)

Reference: Otvos JD, Shalaurova I, May HT, et al. Multimarkers of metabolic malnutrition and inflammation and their association with mortality risk in cardiac catheterisation patients: a prospective, longitudinal, observational, cohort study. Lancet Heal Longev. 2023;4:e72-e82.
Note. Citr = citrate; Ileu = isoleucine; IVX = inflammation vulnerability index; Leu = leucine; MMX = metabolic malnutrition index; MVX = metabolic vulnerability index; S-HDLP = small HDL particle number; Val = valine.

Supplementary Table S3. Multiple Comparisons Results for Multivariate Models Only

Multivariate Model 2		p-value (Original)	p-value (Bonferroni)	p-value (Holm)	p-value (BH)
	**MVX SCORE**				
1	Subclinical hypo	0.146	1.000	1.000	0.399
2	LnTSH	0.137	1.000	1.000	0.399
3	FT4	0.856	1.000	1.000	0.906
4	LnFT3	0.011	0.198	0.198	0.153
5	LnFT3:FT4 ratio	0.047	0.846	0.752	0.270
	Positive TPOAb	0.596	1.000	1.000	0.788
	**MMX SCORE**				
6	Subclinical hypo	0.155	1.000	1.000	0.399
7	LnTSH	0.517	1.000	1.000	0.776
8	FT4	0.684	1.000	1.000	0.821
9	LnFT3	0.354	1.000	1.000	0.637
10	LnFT3:FT4 ratio	0.507	1.000	1.000	0.776
	Positive TPOAb	0.765	1.000	1.000	0.861
	**IVX SCORE**				
11	Subclinical hypo	0.304	1.000	1.000	0.608
12	LnTSH	0.187	1.000	1.000	0.421
13	FT4	0.999	1.000	1.000	0.999
14	LnFT3	0.017	0.306	0.289	0.153
15	LnFT3:FT4 ratio	0.060	1.000	0.900	0.270
	Positive TPOAb	0.613	1.000	1.000	0.788

Note. BH: Benjamini–Hochberg.

Supplementary Table S4. Association of thyroid-related parameters with MVX, MMX and IVX scores in individuals with and without cardiometabolic diseases.

	No cardiometabolic diseases (n=1284)		At least one cardiometabolic disease (n=2695)	
	**B (95% CI)**	**p-value**	**B (95% CI)**	**p-value**
**MVX SCORE**				
Subclinical hypo	0.01 (-0.16;0.18)	0.899	0.10 (-0.02;0.22)	0.094
LnTSH	0.05 (-0.09;0.19)	0.515	0.05 (-0.05;0.15)	0.333
FT4	0.13 (-0.19;0.45)	0.438	0.03 (-0.18;0.24)	0.748
LnFT3	-0.67 (-2.51;1.17)	0.475	-1.56 (-2.86;-0.25)	0.019
LnFT3:FT4 ratio	-0.83 (-2.49;0.83)	0.326	-1.12 (-2.19;-0.06)	0.039
Positive TPOAb	0.08 (-0.09;0.25)	0.349	-0.00 (-0.12;0.12)	0.955
**MMX SCORE**				
Subclinical hypo	0.01 (-0.17;0.19)	0.927	0.11 (-0.01;0.23)	0.071
LnTSH	-0.05 (-0.20;0.10)	0.518	0.05 (-0.04;0.15)	0.272
FT4	0.04 (-0.29;0.38)	0.796	0.05 (-0.16;0.26)	0.617
LnFT3	-1.34 (-3.28;0.59)	0.174	0.18 (-1.12;1.48)	0.782
LnFT3:FT4 ratio	-0.89 (-2.63;0.85)	0.315	0.02 (-1.05;1.08)	0.977
Positive TPOAb	0.06 (-0.12;0.24)	0.510	-0.01 (-0.12;0.11)	0.940
**IVX SCORE**				
Subclinical hypo	0.01 (-0.16;0.19)	0.866	0.07 (-0.05;0.19)	0.277
LnTSH	0.08 (-0.06;0.22)	0.274	0.03 (-0.07;0.13)	0.616
FT4	0.13 (-0.19;0.45)	0.438	0.01 (-0.20;0.23)	0.906
LnFT3	0.01 (-1.85;1.86)	0.996	-1.91 (-3.24;-0.58)	0.005
LnFT3:FT4 ratio	-0.44 (-2.10;1.22)	0.604	-1.31 (-2.39;-0.22)	0.019
Positive TPOAb	-0.00 (-0.12;0.12)	1.000	0.07 (-0.10;0.24)	0.426

Model adjustment by age, sex, race, smoking, diabetes, hypertension, BMI, dyslipidemia, estimated glomerular filtration rate, family history of cardiovascular disease, alcohol intake, physical activity and diet.

Supplementary Table S5. Non-linear association of squared thyroid-related parameters with MVX, MMX and IVX scores in individuals with euthyroid and subclinical hypothyroidism.

	Main analysis		Euthyroid only		Men		Women	
	**B (95% CI)**	**p-value**	**B (95% CI)**	**p-value**	**B (95% CI)**	**p-value**	**B (95% CI)**	**p-value**
**MVX SCORE**								
Sqrt_LnTSH	-1.11 (-2.60;0.37)	0.142	-0.32 (-2.74;2.11)	0.799	-1.35 (-3.55;0.84)	0.227	-1.01 (-3.03;1.02)	0.330
Sqrt_FT4	0.02 (-0.57;0.62)	0.940	0.09 (-0.51;0.70)	0.765	0.83 (-0.19;1.84)	0.110	-0.37 (-1.11;0.37)	0.330
Sqrt_LnFT3	-9.18 (-30.11;11.76)	0.390	-5.39 (-26.96;16.17)	0.624	-84.34 (-131.01;-37.66)	<0.001	5.02 (-18.13;28.17)	0.671
Sqrt_LnFT3:FT4 ratio	-7.46 (-18.82;3.89)	0.198	-6.40 (-18.00;5.21)	0.280	-27.99 (-52.13;-3.85)	0.023	0.47 (-12.17;13.12)	0.942
**MMX SCORE**								
Sqrt_LnTSH	-1.13 (-2.65;0.40)	0.147	0.39 (-2.09;2.87)	0.757	-1.13 (-3.19;0.92)	0.280	-1.28 (-3.54;0.98)	0.268
Sqrt_FT4	-0.06 (-0.66;0.55)	0.855	-0.03 (-0.65;0.59)	0.927	0.65 (-0.30;1.60)	0.179	-0.36 (-1.19;0.47)	0.399
Sqrt_LnFT3	17.18 (-4.28;38.63)	0.117	17.97 (-4.05;39.98)	0.110	-29.60 (-73.49;14.29)	0.186	23.17 (-2.66;49.00)	0.079
Sqrt_LnFT3:FT4 ratio	-13.44 (-25.07;-1.81)	0.024	-12.74 (-24.58;-0.90)	0.035	-48.11 (-70.66;-25.57)	<0.001	-1.63 (-15.75;12.50)	0.822
**IVX SCORE**								
Sqrt_LnTSH	-0.84 (-2.36;0.68)	0.278	-0.57 (-3.04;1.90)	0.652	0.23 (-0.15;0.60)	0.349	0.10 (-0.25;0.45)	0.518
Sqrt_FT4	0.09 (-0.52;0.69)	0.782	0.16 (-0.46;0.77)	0.617	0.69 (-0.35;1.72)	0.193	-0.24 (-0.99;0.52)	0.538
Sqrt_LnFT3	-19.26 (-40.57;2.06)	0.077	-15.35 (-37.33;6.62)	0.171	-87.25 (-134.81;-39.70)	<0.001	-4.97 (-28.47;18.53)	0.679
Sqrt_LnFT3:FT4 ratio	-2.22 (-13.79;9.34)	0.707	-1.35 (-13.18;10.48)	0.823	-10.48 (-35.08;14.11)	0.404	1.79 (-11.04;14.63)	0.784

Model adjusted for age, sex, race, smoking, diabetes, hypertension, BMI, dyslipidemia, estimated glomerular filtration rate, family history of cardiovascular disease, alcohol intake, physical activity, diet, and linear and quadratic (sqrt) terms.

Supplementary Figure S1. Graphs for non-linear associations between FT3:FT4 ratio and MMX.

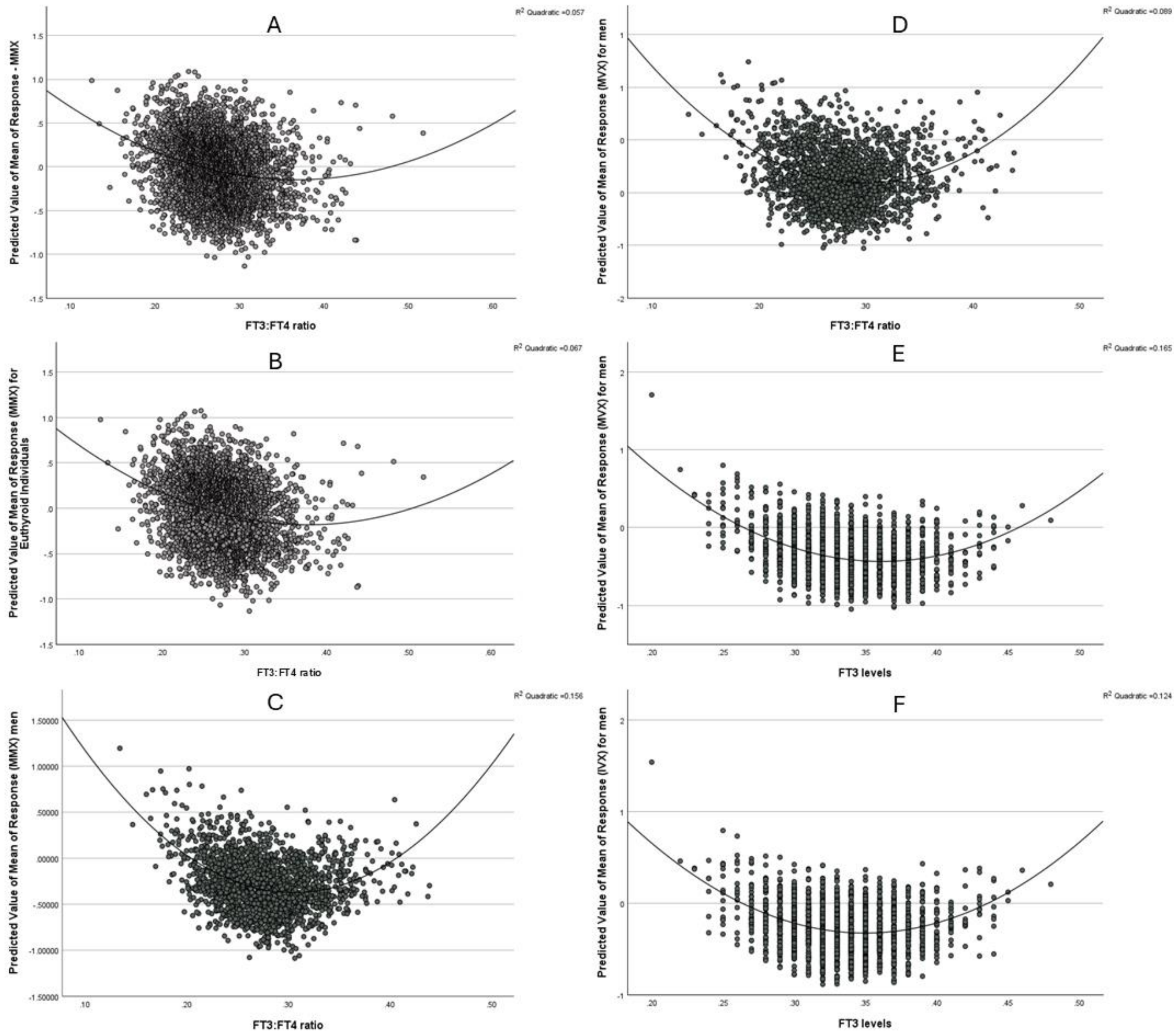

Note: A significant quadratic relationship was observed between FT3:FT4 ratio and MMX in the main analysis (A), for euthyroid individuals (B), and for men (C). For men, there were also significant quadratic relationships between FT3:FT4 ratio and MVX (D), FT3 levels and MVX (E), and FT3 levels and IVX (F).
